# Supplementary material for: Intracellular Metabolomics Switching Alters Extracellular Acid Production and Insoluble Phosphate Solubilization Behavior in Penicillium oxalicum
Source: Metabolites. 2020 Oct 31;10(11):441. doi: 10.3390/metabo10110441 (PMC7692655; doi:10.3390/metabo10110441)
Supplement: Supplementary file 1 [file metabolites-10-00441-s001.pdf]

Intracellular Metabolomics Switching Alters Extracellular Acid Production and Insoluble Phosphate Solubilization Behavior in *Penicillium oxalicum*

Yifan Jiang [1], Fei Ge [1], Feng Li [1], Dayi Zhang [2], Songqiang Deng [3] and Jiang Tian [1,*]

[1] Department of Environment, College of Environment and Resources, Xiangtan University, Xiangtan 411105, China; jyfcjzx@163.com (Y.J.); gefei@xtu.edu.cn (F.G.); Lifeng6220@xtu.edu.cn (F.L.)
[2] School of Environment, Tsinghua University, Beijing 100084, China; zhangdayi@tsinghua.edu.cn
[3] Research Institute for Environmental Innovation (Tsinghua-Suzhou), Suzhou 215009, China; izzydeng1987@163.com
* Correspondence: tianjiangjames23@xtu.edu.cn

Received: 27 September 2020; Accepted: 29 October 2020; Published: date

Table of Contents

Supplementary Materials and Methods 3

Table S1. Metabolites with *p* threshold value 0.05 were identified by one-way ANOVA and Tukey's HSD post-hoc analysis. 7

Table S2. Acidity coefficients of organic acids in three treatments. 10

Figure S1. Maximal biomass (A) and organic acids concentrations (B) of *P. oxalicum*. 11

Figure S2. One-way ANOVA changes of all peaks in three treatments. 12

Figure S3. Total ion chromotagraphy (TIC) of three QC samples in ESI. 13

Figure S4. Relative analysis results of three QC samples in ESI. 14

Figure S5. Non-targeted metabolite profiling analysis of *P. oxalicum*. 15

Figure S6. Heatmap of intracellular metabolites in *P. oxalicum*. 16

Figure S7. Biomarker metabolites and pathways selection.. 17

Figure S8. Intracellular metabolic pathways regulated by P sources in *P. oxalicum*.. 18

Figure S9. Growth condition (A), photomicrograph (B) and Phylogenetic dendrogram. 19

Figure S10. Standard curve of all the detected organic acids in *Penicillium oxalicum*. 20

References 21

Supplementary Materials and Methods

Intracellular Metabolomics and Profiling

Freeze-dried extracted samples were resuspended in 100 μL of methanol/acetonitrile (1:1, v/v) and analyzed by an ultra-HPLC (UHPLC, 1290 Infinity LC, Agilent Technologies, USA) coupled to a quadrupole time-of-flight mass spectrometer (Q-TOF, AB Sciex TripleTOF 6600, USA) for hydrophilic interaction liquid chromatography (HILIC) separation. Untargeted metabolomics analysis was used to identify whole intracellular metabolites in *P. oxalicum*. For high-throughput metabolic profiling, all experimental and QC samples were monitored in both Electron Spray Ionization (ESI) positive and negative modes.

Extraction and identification of extracellular acids

The samples were then analyzed using an HPLC system (Agilent 1260 infinity, USA) equipped with a variable wavelength HPLC detector G1314F and a Platisil ODS C_{18} column (4.6 mm × 250 mm, 5 μm). The mobile phase was acetonitrile/ammonium dihydrogen phosphate (2:98, v/v) injected at a flow rate of 0.5 mL/min and detected at 230 nm. For IP samples, the mobile phase was optimized as methanol /0.1% orthophosphoric acid (2.5:97.5, v/v) at 210 nm to recognize the main peaks. The sample injection volume was 20 μL. The concentrations of organic acids secreted by PSF were calculated on the basis of the peak areas from the calibration curve, which fitted well with linear correlation within the range of 10-100 mg/L (R^2>0.995, Fig. S2).

Quality control of intracellular metabolites

The quality control samples were harvested by the uniform mixing of three experimental samples. In brief, after rapid filtration with subsequent NaCl washing, 10 g globular mycelium of all the eight repeats in TP, IP and control treatment were mixed in triplicate by vortex shocking for 30 min in an ice bath. Afterward, the QC samples were quenched by vortex shocking and cryogenic ultrasonic extraction, and precipitated and centrifuged to eliminate the cellular proteins and lysates. The QC samples were used through the whole programs to estimate the systematic stability of pre- sample injection and LC-MS detecting procedure (Benton et al., 2015).

UHPLC analysis

All the experimental and QC samples were performed using HILIC separation equipped with a 2.1 mm × 100 mm ACQUIY UPLC BEH 1.7 μm column (Waters, Ireland) both in ESI positive and negative modes. The mobile phase A contained 25 mM ammonium acetate and 25 mM ammonium hydroxide in water, mobile phase B contained acetonitrile. The gradient elution program was followed a slight modified protocol of Zhao (Zhao et al., 2019): 85% phase B for 1 min and linearly reduced to 65% in 13 min, and then linearly reduced to 40% in 2 min and kept for 2 min, and then linearly increased from 40% to 95% in 0.1 min, with a 5 min re-equilibration period employed. All the samples were analysis in the automatic sampler which kept in 4 °C through the whole program. Using consistent analysis of samples in random order to avoid the influences that are caused by the signal

fluctuations through instrumental detecting procedures. The QC samples were inserted regularly and analyzed in every eight samples.

Q-TOF analysis

After HILIC separation, Q-TOF (AB Sciex TripleTOF 6600, USA) was used in untargeted metabolomics analysis to identify whole intracellular metabolites in *P. oxalicum* both in ESI positive and negative modes. The ESI source conditions were set as follows a slight modified protocol of Lin. et al[3]. Ion Source Gas1 was set as 60; Ion Source Gas2 was set as 60; curtain gas was 30; source temperature was 600 °C; IonSpray Voltage Floating (ISVF) ± 5500 V (both ESI positive and negative modes); In MS only acquisition, the instrument was set to acquire over the m/z range 60-1000 Da, and the accumulation time for TOF MS scan was set at 0.20 s/spectra. In auto MS/MS acquisition, the instrument was set to acquire over the m/z range 25-1000 Da, and the accumulation time for product ion scan was set at 0.05 s/spectra. Information dependent acquisition (IDA) with high sensitivity mode was selected to acquire the product ion scan. The collision energy (CE) was fixed at 35 V with ± 15 eV; declustering potential (DP) was set at 60 V (+) and −60 V (−). The IDA procedure was set as 4 Da exclude isotopes and 10 candidate ions to monitor per cycle. The QC samples were inserted regularly and analyzed in every eight samples.

MS data analysis

Original MS data were converted to MzXML files using ProteoWizard MSConvert before importing into XCMS for peak alignment, retention time calibration and peak

areas integration. For peak picking, the following parameters were used: centWave m/z = 25 ppm, peakwidth = c (10, 60), prefilter = c (10, 100). For peak grouping, the following parameters were used: bw = 5, mzwid = 0.025, minfrac = 0.5[(Zhao et al., 2019)]. Collection of Algorithms of MEtabolite pRofile Annotation was used for annotation of isotopes and adducts (Benton et al., 2015). In the extracted ion features, only the variables having more than 50% of the nonzero measurement values in at least one group were kept. Compound identification of metabolites was performed by comparing of accuracy m/z value (<25 ppm), and available authentic standards of MS/MS spectra with established database in Shanghai Applied Protein Technology Institute.

Table S1. Metabolites with *p* threshold value 0.05 were identified by one-way ANOVA and Tukey's HSD post-hoc analysis.

No.	Metabolites	F.value	P.value	$-\log_{10}$(P. value)	Tukey's HSD
1	Homoveratric acid	85.73	7.91×10^{-11}	10.10	IP-C; TP-IP
2	D-Fructose	58.11	2.76×10^{-9}	8.56	TP-C; TP-IP
3	R-2-Hydroxycaprylic acid	54.87	4.58×10^{-9}	8.34	IP-C; TP-IP
4	Oxalate	54.05	5.23×10^{-9}	8.28	IP-C; TP-C; TP-IP
5	2'-O-Methyluridine	52.99	6.22×10^{-9}	8.21	IP-C; TP-C; TP-IP
6	Genistein	40.06	6.81×10^{-9}	7.17	IP-C; TP-C; TP-IP
7	1-Methylxanthine	39.67	7.37×10^{-8}	7.13	IP-C; TP-IP
8	D-Tagatose	37.83	1.09×10^{-7}	6.96	TP-C; TP-IP
9	Daidzein	30.00	6.99×10^{-7}	6.16	IP-C; TP-IP
10	4-Pyridoxic acid	29.38	8.21×10^{-7}	6.09	IP-C; TP-IP
11	Apigenin	27.87	1.23×10^{-6}	5.91	IP-C; TP-C; TP-IP
12	p-Cresol	26.63	1.74×10^{-6}	5.76	IP-C; TP-IP
13	2-Hydroxyphenylacetic acid	25.18	2.64×10^{-6}	5.58	IP-C; TP-IP
14	Thymidine	23.45	4.46×10^{-6}	5.35	IP-C; TP-C
15	L-Malic acid	23.31	4.66×10^{-6}	5.33	IP-C; TP-C
16	Pyridoxal Vitamin B6	23.24	4.76×10^{-6}	5.32	IP-C; TP-IP
17	Ethosuximide	22.06	6.91×10^{-6}	5.16	IP-C; TP-C
18	N-Acetylglucosamine 1-phosphate	21.35	8.71×10^{-6}	5.06	IP-C; TP-IP
19	Uracil	20.98	9.84×10^{-6}	5.01	IP-C; TP-C
20	2-Methylbenzoic acid	20.40	1.20×10^{-5}	4.92	IP-C; TP-IP
21	Ribothymidine	20.25	1.26×10^{-5}	4.90	IP-C; TP-IP
22	S-2-Hydroxyglutarate	18.75	2.13×10^{-5}	4.67	IP-C; TP-IP
23	DL-2-Aminoadipic acid	17.59	3.25×10^{-5}	4.49	IP-C; TP-C
24	Alpha-D-Glucose	17.45	3.43×10^{-5}	4.46	TP-C; TP-IP
25	L-Saccharopine	16.87	4.27×10^{-5}	4.37	IP-C; TP-C
26	L-Glutamine	16.37	5.19×10^{-5}	4.29	IP-C; TP-C
27	DL-Serine	16.09	5.80×10^{-5}	4.24	IP-C; TP-C
28	S-Methyl-5'-thioadenosine	14.70	0.000102	3.99	TP-C; TP-IP

29	cis-Aconitate	14.41	0.000115	3.94	TP-C; TP-IP
30	Phosphorylcholine	14.36	0.000118	3.93	IP-C; TP-C
31	L-Pyroglutamic acid	13.77	0.000151	3.82	IP-C; TP-C
32	Kaempferol	13.52	0.000168	3.77	IP-C; TP-IP
33	L-Glutamate	13.47	0.000173	3.76	IP-C; TP-C
34	3-Hydroxycoumarin	12.37	0.000281	3.55	IP-C; TP-IP
35	3,4-Dihydroxybenzoate Protocatechuic acid	11.47	0.00043	3.37	IP-C; TP-IP
36	sn-Glycerol 3-phosphoethanolamine	11.11	0.000512	3.29	IP-C; TP-C
37	UDP-N-acetylglucosamine	10.79	0.000599	3.22	IP-C; TP-C
38	Glycyl-L-leucine	10.61	0.000654	3.18	IP-C; TP-C
39	L-Valine	10.28	0.000772	3.11	IP-C; TP-C
40	Adenosine 3'-monophosphate	10.17	0.000814	3.09	TP-C; TP-IP
41	Citrate	9.86	0.000957	3.02	TP-IP
42	Succinate	9.65	0.001066	2.97	IP-C; TP-C
43	Mevalonic acid	9.38	0.001226	2.91	IP-C; TP-IP
44	D-Arabinono-1,4-lactone	9.38	0.001229	2.91	IP-C; TP-IP
45	Propionic acid	9.25	0.001315	2.88	IP-C; TP-C
46	Phenyllactic acid	8.81	0.001669	2.78	IP-C; TP-IP
47	L-Methionine	8.73	0.001737	2.76	TP-C
48	L-Tyrosine	8.43	0.002051	2.69	IP-C; TP-C
49	Deoxyadenosine	8.42	0.002063	2.69	TP-C
50	L-Gulonic gamma-lactone	8.18	0.002363	2.63	TP-IP
51	L-Arginine	8.08	0.002495	2.60	IP-C; TP-C
52	Sulfaphenazole	7.45	0.003595	2.44	IP-C; TP-IP
53	Guanosine	7.21	0.00414	2.38	IP-C
54	Glyceric acid	7.04	0.004561	2.34	IP-C; TP-C
55	Pseudouridine	6.99	0.004718	2.33	IP-C; TP-IP
56	L-Iditol	6.87	0.005056	2.30	IP-C; TP-C
57	L-Ribulose	6.74	0.005476	2.26	IP-C; TP-C
58	L-Lysine	6.60	0.005978	2.22	IP-C; TP-C
59	Hydroxyisocaproic acid	6.43	0.006634	2.18	IP-C; TP-IP
60	Pantetheine	6.43	0.006634	2.18	IP-C; TP-C

61	Glutathione disulfide	6.19	0.00771	2.11	IP-C; TP-C
62	D-Lyxose	6.08	0.008268	2.08	TP-IP
63	Adenosine	5.58	0.01142	1.94	IP-C
64	Uridine	5.39	0.012873	1.89	IP-C
65	m-Chlorohippuric acid	4.95	0.017294	1.76	IP-C; TP-C
66	Adenosine monophosphate AMP	4.86	0.018406	1.74	TP-C; TP-IP
67	L-Leucine	4.78	0.019415	1.71	IP-C
68	DL-lactate	4.76	0.019685	1.71	IP-C
69	Dulcitol	4.60	0.022089	1.66	TP-IP
70	Cytidine	4.36	0.026095	1.58	TP-IP
71	Anthranilic acid Vitamin L1	4.36	0.026118	1.58	IP-C; TP-IP
72	Norethindrone Acetate	4.24	0.028444	1.55	TP-C
73	3,3',4,5'-Tetrahydroxy-trans-stilbene	3.85	0.037706	1.42	IP-C

2

Table S2. Acidity coefficients of organic acids in three treatments.

Treatment	Organic acids	pK_a
TP	Oxalic acid	1.27
	Gluconic acid	3.86
	Pyruvic acid	2.39
	Fumaric acid	2.18
IP	Oxalic acid	1.27
	Malic acid	3.46
	Gluconic acid	3.86
C	Gluconic acid	3.86
	Acetic acid	4.76
	Tartaric acid	3.04

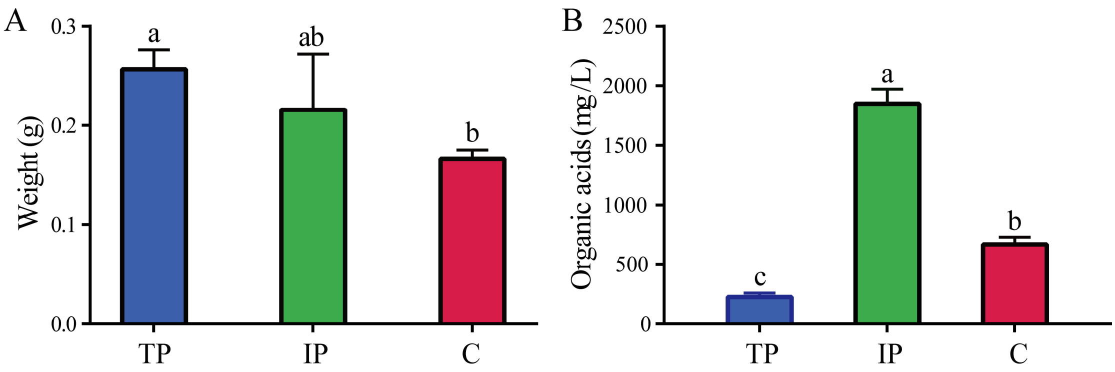

Figure S1. Maximal biomass (**A**) and organic acids concentrations (**B**) of *P. oxalicum* in PKV medium with TP, IP, or C as the sole P source. Data are the average result of three replicates with standard deviations. Different letters above the columns indicate a significant difference between the expression levels of a gene under different growth conditions ($p < 0.05$).

Figure S2. One-way ANOVA changes of all peaks in three treatments in both ESI positive (**A**) and negative (**B**) mode. Important features selected by one-way ANOVA and Tukey's HSD with p value threshold 0.05. Data represent $-\log_{10}$(raw p-value) changes. Dotted line represent the threshold p = 0.05.

Figure S3. Total ion chromotagraphy (TIC) of three QC samples in ESI positive (**A**) and negative (**B**) mode. The good repeatability of three QC samples in both positive and negative mode represent the good stability of whole UHPLC-Q-TOF detecting program.

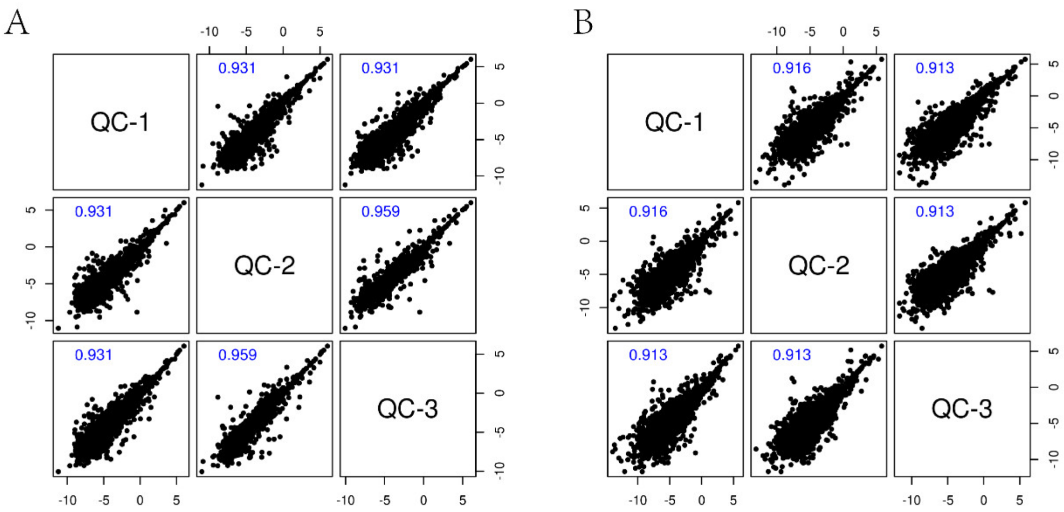

Figure S4. Relative analysis results of three QC samples in ESI positive (**A**) and negative (**B**) mode. Pearson relative analysis was used to detect the intensity values. X and Y axil represent the numerical intensity values, respectively. The correlation coefficient results (blue) were accounted by the sum of RSD (≤30%) of QC intensity values divide sum of total QC intensity values, demonstrating the good repeatability of QC samples and stability of whole detecting program.

Figure S5. Non-targeted metabolite profiling analysis of *P. oxalicum* in response of three P sources by PCA (A), PLS-DA (B) and HCA (C). **A**: Scores plot between the selected Principle components (PCs). The explained variances of three treatments KH_2PO_4 (control, red), $Ca_3(PO_4)_2$ (TP, blue) and $FePO_4$ (IP, green) are shown in brackets. PC1 and PC2 represent approximately 37.2% and 23.2% of total variance of the data set, respectively. **B**: Partial lease squares discriminant analysis (PLS-DA) scores plots discriminating among the three P sources of control KH_2PO_4 (red), $Ca_3(PO_4)_2$ (blue) and $FePO_4$ (green) with the total variance of 30.8% and 25.2% of the data set, respectively. **C**: Hierarchical analysis (HCA) plot using Pearson's method showing the differences in the metabolic response of P sources of KH_2PO_4 (red), $Ca_3(PO_4)_2$ (blue) and $FePO_4$ (green).

Figure S6. Heatmap of intracellular metabolites in *P. oxalicum* in Insol-P and Sol-P treated medium arranged by chemical properties using Pearson's average clustering algorithm. Z-score for each feature was used to color the map from absent or low concentration metabolites (green) to high concentration metabolites (red).

Figure S7. Biomarker metabolites and pathways selection. **A**. Important features selected by volcano plot with fold change (FC) threshold (×) 2 and t-test threshold (y) 0.1. The red and yellows circles represent features above the FC threshold of +2 and -2, respectively. **B**. OPLS-DA loadings S-plot showing the variable importance in a model, combing the covariance (p[1]) and the correlations (p(corr)) loading profile. Cycles in different colors represent various p[1] and p(corr) values. **C**. Intracellular metabolomics pathway analysis in P. oxalicum with Insol-P treated compare to Sol-P treat medium according to KEGG pathway libraries.

Figure S8. Intracellular metabolic pathways regulated by P sources in *P. oxalicum*. Identified metabolites from Q-TOF are marked with colored circles. Briefly, blue, red, purple and yellow circles represent the metabolite intensity of the samples that affected by Insol-P vs Sol-P, IP vs TP, TP vs Sol-P, IP vs Sol-P, respectively. The diameters of the cycles represent the intensities of the intracellular metabolites in four comparisons. The solid circles represent the significant increase (FC > 2 fold, $p < 0.05$) and decrease ($\log_2(FC) < -1$, $p < 0.05$) intensities of important metabolites comparing to each control treatments. The hollow circles represent no significant changes ($0.5 < FC < 2$, $p < 0.05$) of the metabolic intensities. Solid lines show the intracellular metabolic pathways that exist in *P. oxalicum*, while the dotted lines show the supposed metabolic pathways.

Fig. S9

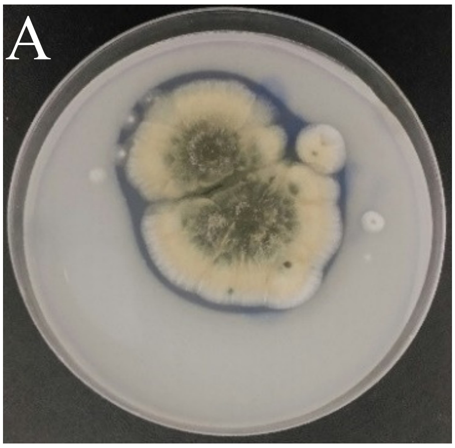

0.045mm

C

Figure S9. Growth condition (**A**), photomicrograph (**B**) and Phylogenetic dendrogram of *Penicillium oxalicum* strain PSF-4 (**C**). Bootstrap values (%) are indicated at the nodes.

Figure S10. Standard curve of all the detected organic acids in *Penicillium oxalicum* analyzed by HPLC. A: the order of concentration from bottom to top is 1, 2, 5, 8 and 10 mM; B-G: the order of concentration from bottom to top is 10, 20, 50, 80 and 100 mg/L.

References

Benton, HP.; Ivanisevic, J.; Mahieu, NG.; Kurczy, ME.; Johnson, CH.; Franco, L. Autonomous metabolomics for rapid metabolite identification in global profiling. *Anal. Chem*. **2015**. *87*, 884-891. https://doi.org/10.1021/ac5025649.

Zhao, Y.; Nie, S.; Yi, M.; Wu, N.; Wang, W.; Zhang, Z. UPLC-QTOF/MS-based metabolomics analysis of plasma reveals an effect of Xue-Fu-Zhu-Yu capsules on blood-stasis syndrome in CHD rats. *J. Ethnopharmacol*. **2019**, *241*, 111908. https://doi.org/10.1016/j.jep.2019.111908.
